# Prognostic value of patient-reported quality of life for survival in oesophagogastric cancer: analysis from the population-based POCOP study

**DOI:** 10.1007/s10120-021-01209-1

**Published:** 2021-07-12

**Authors:** J. J. van Kleef, W. P. M. Dijksterhuis, H. G. van den Boorn, M. Prins, R. H. A. Verhoeven, S. S. Gisbertz, M. Slingerland, N. Haj Mohammad, G.-J. Creemers, K. J. Neelis, J. Heisterkamp, C. Rosman, J. P. Ruurda, E. A. Kouwenhoven, L. V. van de Poll-Franse, M. G. H. van Oijen, M. A. G. Sprangers, H. W. M. van Laarhoven, On behalf of the Dutch Upper GI Cancer Group (DUCG)

**Affiliations:** 1 Department of Medical Oncology, Amsterdam UMC, University of Amsterdam, Cancer Center Amsterdam, Office D3-312, PO Box 22660, 1100DD Amsterdam, The Netherlands; 2 Department of Medical Psychology, Amsterdam UMC, University of Amsterdam, Public Health Research Institute, Amsterdam, The Netherlands; 3 Department of Research and Development, Netherlands Comprehensive Cancer Organisation (IKNL), Utrecht, The Netherlands; 4 Department of Surgery, Amsterdam UMC, University of Amsterdam, Cancer Center Amsterdam, Amsterdam, The Netherlands; 5 Department of Medical Oncology, Leiden University Medical Center, Leiden, The Netherlands; 6 Department of Medical Oncology, University Medical Center Utrecht, Utrecht University, Utrecht, The Netherlands; 7 Department of Medical Oncology, Catharina Hospital, Eindhoven, The Netherlands; 8 Department of Radiotherapy, Leiden University Medical Center, Leiden, The Netherlands; 9 Department of Surgery, Elizabeth-TweeSteden Hospital, Tilburg, the Netherlands; 10 Comprehensive Cancer Network EMBRAZE, Breda, The Netherlands; 11 Department of Surgery, Radboud University Medical Center, Nijmegen, The Netherlands; 12 Department of Surgery, University Medical Center Utrecht, Utrecht, The Netherlands; 13 Department of Surgery, Hospital Group Twente, Almelo, The Netherlands; 14 Department of Psychosocial Research and Epidemiology, The Netherlands Cancer Institute, Amsterdam, The Netherlands; 15 Department of Medical and Clinical Psychology, Center of Research on Psychological and Somatic Disorders (CoRPS), Tilburg University, Tilburg, The Netherlands

**Keywords:** Quality of life, Prognostic, Survival, Oesophageal cancer, Gastric cancer, Population based

## Abstract

**Background:**

Accumulating evidence of trials demonstrates that patient-reported health-related quality of life (HRQoL) at diagnosis is prognostic for overall survival (OS) in oesophagogastric cancer. However, real-world data are lacking. Moreover, differences in disease stages and tumour-specific symptoms are usually not taken into consideration. The aim of this population-based study was to assess the prognostic value of HRQoL, including tumour-specific scales, on OS in patients with potentially curable and advanced oesophagogastric cancer.

**Methods:**

Data were derived from the Netherlands Cancer Registry and the patient reported outcome registry (POCOP). Patients included in POCOP between 2016 and 2018 were stratified for potentially curable (cT1-4aNallM0) or advanced (cT4b or cM1) disease. HRQoL was measured with the EORTC QLQ-C30 and the tumour-specific OG25 module. Cox proportional hazards models assessed the impact of HRQoL, sociodemographic and clinical factors (including treatment) on OS.

**Results:**

In total, 924 patients were included. Median OS was 38.9 months in potentially curable patients (*n* = 795) and 10.6 months in patients with advanced disease (*n* = 129). Global Health Status was independently associated with OS in potentially curable patients (HR 0.89, 99%CI 0.82–0.97), together with several other HRQoL items: appetite loss, dysphagia, eating restrictions, odynophagia, and body image. In advanced disease, the Summary Score was the strongest independent prognostic factor (HR 0.75, 99%CI 0.59–0.94), followed by fatigue, pain, insomnia and role functioning.

**Conclusion:**

In a real-world setting, HRQoL was prognostic for OS in patients with potentially curable and advanced oesophagogastric cancer. Several HRQoL domains, including the Summary Score and several OG25 items, could be used to develop or update prognostic models.

**Supplementary Information:**

The online version contains supplementary material available at 10.1007/s10120-021-01209-1.

## Introduction

The prognostic value of health-related quality of life (HRQoL) on overall survival (OS) has been described in patients with several types of cancer [1–4], including oesophagogastric cancer [3, 5–10]. Most knowledge regarding the prognostic value of HRQoL in oesophagogastric cancer originates from RCTs [3, 5, 6, 8–10] rather than from population-based studies [7]. As the typical trial patient reflects only 5–10% of the patient population due to stringent inclusion criteria of RCTs, trial populations may not adequately represent the real-world cancer population [11, 12]. Moreover, the prognostic value of HRQoL may vary between patients with potentially curable and advanced (i.e., irresectable or metastatic) oesophagogastric cancer. In patients with advanced oesophagogastric cancer participating in RCTs, an association between fatigue [10], reflux [10], social functioning [6], physical functioning [13] and overall survival (OS) was found, while physical symptoms [9] were prognostic for OS in potentially curable patients. Since the majority of the patient reported outcome (PRO) data were collected in patients with advanced disease, results of potentially curable patients are scarce. Population-based data could add valuable information to those collected in RCTs on the prognostic value of HRQoL in both patient subgroups.

The European Organisation for Research and Treatment of Cancer (EORTC) QLQ-C30 is the most commonly used questionnaire to measure HRQoL in oesophagogastric cancer [14]. It can be supplemented by the QLQ-OG25 questionnaire–a module assessing typical symptoms within oesophagogastric cancer [15]. To our knowledge, the prognostic value of the OG25 module has not been studied yet. In addition, the QLQ-C30 Summary Score was recently developed, and combines scores of symptom and functioning scales of the QLQ-C30 into a single score [16]. While recent results of a population-based study showed a strong prognostic value of the Summary Score in Dutch patients with colorectal, prostatic and haematological malignancies [17], its prognostic value within oesophagogastric cancer has yet to be determined.

Since 2016, clinical and PRO data of Dutch oesophagogastric cancer patients are collected in the Prospective Observational Cohort Study of Oesophageal-gastric cancer Patients (POCOP), including the QLQ-C30 and OG25 questionnaires [18]. POCOP is a Dutch population-based nationwide cohort of patients diagnosed with oesophagogastric cancer. Within POCOP, PRO data are gathered in close collaboration with academic and peripheral hospitals (covering > 50% of Dutch hospitals). Questionnaires are sent out via PROFILES^19^ offering an online questionnaire sent by e-mail or a paper–pencil questionnaire sent by mail. If needed, a reminder via telephone, mail or e-mail is conducted to increase response rates. Questionnaires are sent before the start of treatment, and after 3, 6, 9, 12, 18, and 24 months, and yearly thereafter. Clinical data and survival data are gathered via the Netherlands Cancer Registry (NCR), in which data from medical charts and municipality registries are registered by trained data mangers. All POCOP data are stored within the NCR and PROFILES databases. Participating hospitals can request data of patients treated in their hospitals. Additionally, research groups can request data for study purposes after filing a research plan to the Dutch Upper GI Cancer Group, as explained more extensively by Coebergh van den Braak et al. [18] Furthermore, patients can request their own data and decide if they want to share it with their treating physician to for example increase patient outcome.

The aims of this population-based study were to assess the independent prognostic value of the recently developed Summary Score, the frequently used Global Health Status (GHS) and the other QLQ-C30 and QLQ-OG25 scales and items on OS in patients with potentially curable (cT1-4a/M0) and advanced (cM1 or cT4b) oesophagogastric cancer in a real-world setting, alongside sociodemographic and clinical prognostic variables.

## Methods

### Design and data source

Clinical data regarding the patient, tumour, and treatment were derived from the nationwide Netherlands Cancer Registry (NCR). Information on vital status was obtained by linkage to the Dutch municipality registry in February 2020. Baseline PROMs data of the included patients were extracted from the POCOP registry [15]. All patients provided written informed consent for study participation and linkage with the NCR.

We included patients who were diagnosed with cancer in the oesophagus, gastro-oesophageal junction or stomach (C15 and C16 according to the 3rd version of the ICD-10 [20]) between 2016 and 2018, to have enough follow-up data on survival times. Patients were eligible for inclusion irrespective of treatment type, but were excluded if the baseline questionnaire was completed more than seven days after start of initial treatment. The baseline PROMs were sent via mail (paper) or email (electronic questionnaire using the PROFILES platform [19]) dependent on patients’ preferences.

### HRQoL

The 30-item QLQ-C30 (v3.0) is a validated cancer-specific questionnaire, to be completed by the patient [21]. It contains five functional scales, a global QoL scale (GHS), three symptom scales and six single items [21]. A scoring procedure was applied according to the EORTC scoring manual [22]. Herewith, scores were linearly transformed to a score between 0 and 100. The QLQ-C30 Summary Score was calculated as the mean of the combined thirteen QLQ-C30 scale and item scores (excluding GHS and financial difficulties). Higher functioning scores, GHS, and Summary Scores indicate better HRQoL, whereas higher symptom scores represent more severe symptoms. The QLQ-OG25 scales and items are scored similarly, in which a higher score represents more severe symptoms.

### Clinical and sociodemographic factors

Clinical and sociodemographic variables included age at diagnosis, marital status, ECOG performance status (PS), body mass index, and weight loss in the month before diagnosis, the presence of peritoneal or liver metastases, number of metastatic sites, number of comorbidities, clinical disease stage, tumour differentiation grade, and treatment type. Selection of these variables was based on a systematic review [5] and clinical data availability in the NCR and POCOP registry [18]. Initial treatments for potentially curable patients, i.e., those with a cT1-4a/M0 disease stage, consisted of: (1) resection (with or without (neo)adjuvant chemotherapy or chemoradiotherapy [CRT; chemotherapy with concurrent long scheme radiotherapy, i.e. ≥ 23 fractions or a duration of ≥ 28 days]), (2) chemoradiotherapy only, i.e., without a resection, and (3) other treatments (systemic treatment, radiotherapy, best supportive care [BSC]). Initial treatments for patients with advanced (i.e., metastatic [cM1] or irresectable [cT4b]) disease consisted of: (1) systemic therapy (chemotherapy and/or targeted therapy with or without radiotherapy, (2) BSC (including radiotherapy and stent placement) or (3) other (resection of primary tumour or metastases).

### Statistical analysis

The primary endpoint was OS defined from the date of diagnosis till the date of death by any cause. OS was calculated from date of diagnosis, because baseline variables were included at diagnosis and patients could enter at any time in the POCOP cohort (after diagnosis, during treatment or during follow-up). Patients alive at the time of analysis were censored at the date of last follow-up (February 1, 2020). Our primary HRQoL variables of interest were the GHS and the novel Summary Score. Functioning and symptoms scales/items were of secondary interest.

The Kaplan–Meier method was used to estimate median survival given the right-censoring of patients (i.e. when patients were still alive at the end of the study). Cox’s proportional hazard regression models were used to assess the impact of HRQoL and other clinical and sociodemographic variables on OS. The hazard ratios (HRs) of all HRQoL scales were reported to represent a clinically meaningful difference of 10 points [23].

To start, a multivariable model with clinical and sociodemographic variables [5] was constructed using backwards selection (starting with full model and removal of variables if *p* > 0.05). To investigate the added value of HRQoL variables, first as part of a pre-selection, univariate analyses were performed to assess the association of single HRQoL variables with OS. Subsequently, in a multivariable model each HRQoL variable was analysed separately from the other HRQoL variables but in combination with clinical and sociodemographic variables. Lastly, for exploratory purposes, a multivariable model was fitted with forced entry of clinical and sociodemographic variables, and multiple HRQoL variables to account for associations among HRQoL scores [7]. HRs of HRQoL items were regarded to be statistically significant at *p* < 0.01. A post hoc power analysis was performed on the primary HRQoL variables of interest (GHS and the Summary Score) to estimate the smallest value of the HR that could be detected given our patient sample, considering 80% power, a 1% alpha-level lowering the risk of type I errors due to multiple testing, the standard deviation, the level of censoring and the squared multiple-correlation coefficient between the HRQoL variable of interest and other clinical and sociodemographic variables in the Cox model. Nagelkerke’s *R*^2^ was used to assess the outcome variance explained by clinical, sociodemographic, and HRQoL variables. An increase of 5% in explained variance by HRQoL variables alongside clinical variables was considered clinically relevant [24]. All analysis were stratified per patient group, i.e., potentially curable versus advanced disease, and performed in Stata 16.1 (StataCorp, College Station, TX).

## Results

In total, of the 1152 POCOP patients who participated in the PRO registry, 924 patients were included and 228 were excluded. Exclusion was based on baseline questionnaires returned > 7 days after starting treatment (defined as baseline non-respondents). OS did not differ significantly between included and excluded patients (log-rank: *p* = 0.42). In addition, the proportion of patients with advanced disease was comparable between included (*n* = 129, 14% and excluded patients (*n* = 39, 17%), suggesting no obvious differences in OS between respondents and non-respondents.

Of all included patients (*n* = 924), 787 (85.2%) completed the baseline questionnaire before the start of treatment and 137 (14.8%) within seven days of starting treatment. Mean GHS scores (73.7 versus 73.6, student’s *t* test, *p* = 0.91) did not differ significantly between patients with a true versus non-true (i.e., within seven days of starting treatment) baseline questionnaire. OS was also comparable (log-rank: *p* = 0.88), suggesting no significant association of questionnaire compliance or worsened GHS scores with early death in the included cohort.

### Patient characteristics

Of the entire cohort, 795 (86%) had potentially curable disease (Table 1). In the potentially curable and advanced subgroup, 277 (34.8%) and 105 (81.4%) patients died, and estimated median survival was 38.9 and 10.6 months, respectively. Potentially curable patients were treated with surgery alone (5.7%), surgery plus CRT (59.9%) surgery plus CT (14%) or CRT alone (17.5%). Twenty-four patients (3%) received systemic therapy, radiotherapy or BSC, due to for example poor PS, interval metastases or on patient’s request. Patients with advanced disease at diagnosis were treated with systemic therapy (59.7%), BSC including radiotherapy, stent and/or pain management (27.9%) and other treatments, e.g., resection of metastases (12.4%).

**Table 1:** Patient, tumour and treatment characteristics

	Patients with potentially curable disease (*n* = 795)		Patients with advanced disease (*n* = 129)	
	No	%	No	%
Age (mean, SD)	66.5 (8.4)	–	65.9 (8.8)	–
Gender				
Male	611	76.9	94	72.9
Female	184	23.1	35	27.1
Performance status				
0–1	653	82.1	92	71.3
2–4	32	4	13	10.1
Unknown	110	13.8	24	18.6
Comborbidities				
0	183	23	37	28.7
1	199	25	34	26.4
≥ 2	235	29.6	33	25.6
Unknown	178	22.4	25	19.4
Weight loss in kilograms (mean, SD)	2.2 (3.6)	–	3.3 (3.9)	–
Tumour location				
Oesophagus	585	73.6	67	51.9
Gastro-oesophageal junction	101	12.7	20	15.5
Stomach	109	13.7	42	32.6
Histology				
Adenocarcinoma	656	82.5	114	88.4
Squamous cell carcinoma	139	17.5	15	11.6
Histological differentiation grade				
1	29	3.7	6	4.7
2	296	37.2	33	25.6
3/4	294	37	52	40.3
Unknown	176	22.1	38	29.5
Clinical stage				
1	65	8.2	–	–
2	202	25.4	–	–
3	396	49.8	2	1.6
4	89	11.2	127	98.5
Unknown	43	5.4	–	–
Number of distant metastatic sites				
0	NA		9	7.0
1			80	62.0
≥ 2			40	31.0
Initial treatment				
Resection (± systemic treatment or chemoradiotherapy)	632	79.5	–	–
Chemoradiotherapy	139	17.5	–	–
Palliative treatment (systemic treatment, radiotherapy or BSC)	24	3	–	–
Palliative systemic treatment (+ /– radiotherapy)	–	–	77	59.7
BSC (± radiotherapy)	–	–	36	27.9
Other	–	–	16	12.4

*SD *standard deviation, *BSC *best supportive care, *NA *not applicable. Potentially curable disease: cT1-4a, N-all, M0; advanced disease: cT4b or M1

### Health-related quality of life

In total, missing HRQoL data on item level (not on patient level) was on average 0.7% and ranged from 0.1% to 1.5% per item. For the potentially curable subgroup, mean symptom scores were highest for anxiety (50.9), eating restrictions (30.8), and fatigue (23.7), see Table 2. For the advanced disease subgroup, mean symptom scores were highest for anxiety (56.7), eating restrictions (40.7), and worrying about weight loss (31.8).

**Table 2:** Mean and standard deviation of baseline HRQoL scores per patient subgroup

EORTC QLQ-C30	Patients with potentially curable disease		Patients with advanced disease	
	Baseline values		Baseline values	
	*n*	Mean (sd)	*n*	Mean (sd)
Summary score	786	84.7 (12.7)	124	80.6 (12.7)
Global health status	790	74.3 (17.8)	127	70.4 (17.3)
Functioning EORTC QLQ-C30				
Physical functioning	794	87.4 (15.8)	128	84.6 (17.1)
Role functioning	794	83 (24.6)	128	76 (25.9)
Emotional functioning	792	77.9 (19.7)	127	74.1 (19.5)
Cognitive functioning	792	89.7 (16.8)	127	89.5 (15.5)
Social functioning	792	85.3 (21.2)	127	80.6 (24.5)
Symptoms EORTC QLQ-C30				
Fatigue	793	23.7 (21.6)	128	30.8 (22.2)
Nausea and vomiting	793	10.7 (18.2)	128	14.2 (20.2)
Pain	794	14.5 (18.9)	129	18.9 (21.3)
Dyspnea	794	11.6 (20.4)	128	13.5 (20.3)
Insomnia	793	22.3 (27.6)	128	25.5 (27.3)
Appetite loss	790	19.4 (27.2)	127	30.7 (33.8)
Constipation	791	12.6 (22.2)	128	16.9 (24.4)
Diarrhea	792	6.5 (16.5)	127	7.3 (17.8)
Financial difficulties	788	6.4 (16.9)	127	6.3 (17.2)
Symptoms EORTC QLQ-OG25				
Dysphagia	793	21.4 (22.9)	125	27.5 (25.1)
Eating restrictions	790	30.8 (27.6)	125	40.7 (31.3)
Reflux	789	6.9 (16.9)	125	6 (12.6)
Odynophagia	787	23.6 (25.7)	125	24.1 (25.1)
Pain and discomfort	788	17.4 (23.6)	125	21.3 (22.7)
Anxiety	791	50.7 (25.8)	127	56.7 (26.3)
Eating with others	788	15.3 (27.8)	122	16.4 (28.2)
Dry mouth	791	13.7 (23.2)	125	19.5 (28.8)
Trouble with taste	789	11.6 (23.5)	124	17.8 (27.4)
Body Image	791	9.3 (20.6)	125	18.1 (26.9)
Trouble swallowing saliva	793	9.7 (21.6)	129	12.9 (25.5)
Choked when swallowing	792	6.7 (17.6)	129	4.9 (13.2)
Coughing	788	20.1 (22.4)	129	18.3 (22)
Trouble talking	789	3.9 (12.5)	127	6 (15.9)
Worrying about weight loss	791	18.9 (26.5)	128	31.8 (31.3)

### Prognostic value of the Summary Score and Global Health Status score

**Fig. 1:**
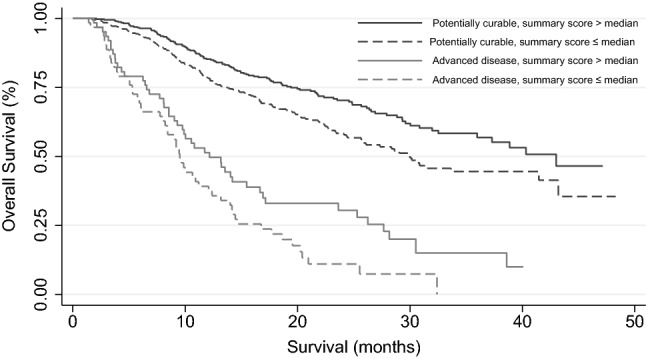
Survival curve stratified per patient subgroup and EORTC QLQ-C30 Summary Score. The black line and black dashed line represent patients with potentially curable disease with a Summary Score above median and below median, respectively. The grey line and dashed grey line represent patients with advanced disease with a Summary Score above median and below median, respectively

Figure 1 shows the association between the Summary Score and OS stratified per patient subgroup (log-rank, *p* = 0.002 and *p* = 0.03 for the potentially curable and advanced disease subgroup, respectively). In the potentially curable subgroup, the Summary Score was only significantly associated with OS in univariable Cox regression analysis, but not in multivariable analysis (Tables 3 and 4). In the advanced disease subgroup, the Summary Score was significantly associated with OS in both uni- and multivariable analysis (Tables 3 and 4). Adjusted for clinical variables, for every 10-point increase in the Summary Score a 25% reduction in the risk of death at any given time was observed (HR 0.75, 99% CI 0.59–0.94, *p* = 0.001).

**Table 3:** Univariate cox regression analysis of EORTC QLQ-C30 Global Health Status and Summary Score

Primary HRQoL variables	Patients with potentially curable disease		Patients with advanced disease	
Univariate cox regression analysis	HR (99% CI)	*p*	HR (99% CI)	*p*
Summary score	0.88 (0.79–0.98)	0.003	0.76 (0.62–0.92)	< 0.001
Global health status	0.89 (0.82–0.96)	< 0.001	0.82 (0.70–0.95)	0.001

*HRQoL *Health-related quality of life, *HR *hazard ratio, hazard ratios are given for every 10-point increase in HRQoL scores

**Table 4:** Multivariate cox regression analysis of EORTC QLQ-C30 and OG25 symptom and functioning scales and items for patients with potentially curable disease

Multivariate cox regression analysis	Patients with potentially curable disease	
Single HRQoL items adjusted for clinical variables	HR (99% CI)	*p*
Summary score	0.95 (0.84–1.06)	0.22
Global health status	**0.89 (0.82–0.97)**	**0.001**
Physical functioning	0.95 (0.87–1.05)	0.17
Role functioning	0.96 (0.91–1.02)	0.09
Nausea and vomiting	1.07 (0.99–1.15)	0.02
Pain	1.07 (0.99–1.16)	0.03
Appetite loss	**1.06 (1.00–1.12)**	**0.01**
Dysphagia	**1.12 (1.05–1.19)**	** < 0.001**
Eating restrictions	**1.10 (1.04–1.16)**	** < 0.001**
Odynophagia	**1.06 (1.01–1.13)**	**0.004**
Trouble with taste	1.04 (0.99–1.09)	0.14
Body image	**1.08 (1.01–1.16)**	**0.002**
Worrying about weight loss	1.04 (0.99–1.08)	0.10

*HRQoL *Health-related quality of life, *HR *hazard ratio. Values in bold were statistically significant at *p* < 0.01. Hazard ratios are given for every 10-point increase in HRQoL scores. Clinical covariates for the potentially curable subgroup were treatment type, clinical stage and tumour differentiation grade

**Fig. 2:**
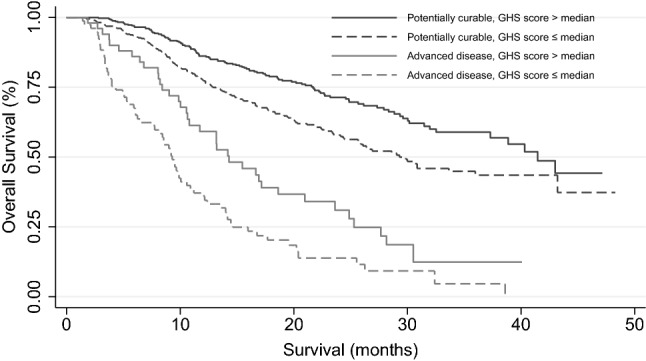
Survival curve stratified per patient subgroup and EORTC QLQ-C30 Global Health Status score. The black line and black dashed line represent patients with potentially curable disease with a Global Health Score above median and below median, respectively. The grey line and dashed grey line represent patients with advanced disease with a Global Health Score above median and below median, respectively

Figure 2 shows the association between GHS and OS stratified per patient subgroup (log-rank: *p* = 0.04 and *p* = 0.005 for the potentially curable and advanced disease subgroup, respectively). In the potentially curable subgroup, Cox regression analysis showed a significant association with OS in both uni- and multivariable analysis. Adjusted for clinical variables, for every 10-point increase in the GHS score an 11% reduction in the risk of death at any given time was observed (HR 0.89, 99% CI 0.82–0.97, *p* < 0.001). In the advanced disease subgroup, GHS was only significantly associated with OS in univariate analysis. This effect did not remain when adjusted for other clinical factors, see Table 4.

### Prognostic value of QLQ-C30 & OG25 symptom and functioning scores

Univariate cox regression analysis for QLQ-C30 and OG25 scales and items are shown in Online Resource 1. In the potentially curable subgroup, multivariable analysis showed that appetite loss, dysphagia, eating restrictions, body image, and odynophagia were independently associated with OS, with adjusted HRs ranging from 1.06 to 1.12 (Table 4). In the advanced disease subgroup, role functioning, fatigue, pain, and insomnia were independently associated with OS, with adjusted HRs ranging from 1.13 to 1.16 for symptom items, and 0.89 for role functioning (Table 5).

**Table 5:** Multivariate cox regression analysis of EORTC QLQ-C30 and OG25 symptom and functioning scales and items for patients with advanced disease

Multivariate cox regression analysis	Patients with advanced disease	
Single HRQoL items adjusted for clinical variables	HR (99% CI)	*p*
Summary score	**0.75 (0.59–0.94)**	**0.001**
Global health status	0.88 (0.75–1.04)	0.05
Physical functioning	0.91 (0.77–1.09)	0.19
Role functioning	**0.89 (0.80–1.00)**	**0.008**
Fatigue	**1.16 (1.03–1.31)**	**0.002**
Nausea and vomiting	1.13 (0.98–1.29)	0.02
Pain	**1.16 (1.02–1.32)**	**0.003**
Insomnia	**1.13 (1.02–1.25)**	**0.002**
Appetite loss	1.08 (1.00–1.18)	0.01
Eating restrictions	1.07 (0.98–1.18)	0.05
Dry mouth	1.07 (0.97–1.18)	0.06
Body image	1.05 (0.95–1.16)	0.21
Worrying about weight loss	1.06 (0.98–1.15)	0.05

*HRQoL* health-related quality of life. *HR*  hazard ratio. Values in bold were statistically significant at *p *< 0.01. Hazard ratios are given for every 10-point increase in HRQoL scores. Clinical covariates for the advanced subgroup were treatment type, performance status, peritoneal metastases, age, and marital status

For exploratory purposes to assess associations between HRQoL items, multiple HRQoL variables were added to the clinical model in which only eating restrictions (HR 1.10, 99% CI 1.04–1.16, *p* < 0.001) remained significantly associated with survival in the potentially curable subgroup and the Summary Score in the advanced disease subgroup (HR 0.75, 99% CI 0.59–0.94, *p* = 0.001).

Post hoc power analysis showed for potentially curable patients that the smallest effect size (HR) of HRQoL variables that could be detected given 80% power and 1% alpha in the multivariable clinical model was 0.90 for GHS and 0.85 for the Summary Score. For patients with advanced disease, a minimal effect size of 0.83 for GHS and 0.77 for the Summary Score was calculated.

### Explained variance

In the potentially curable subgroup, clinical variables (treatment type, clinical stage, differentiation grade) explained 24.4% of the variance in OS. Adding separate HRQoL variables to the clinical model explained 2.5% additionally for GHS, 1.0% for appetite loss, 3.3% for dysphagia, 3.8% for eating restrictions, 2.4% for odynophagia, and 1.8% for body image.

In the advanced disease subgroup, clinical and sociodemographic variables (treatment type, PS, peritoneal metastases, age and marital status) explained 24.6% of the variance in OS. Role functioning explained another 3.6% additionally, fatigue 4.8%, pain 4.1% and insomnia 4.7%. The Summary Score explained most of the OS variance, i.e., 5.6% additionally within this subgroup.

## Discussion

Several studies have reported on the prognostic value of HRQoL in oesophagogastric cancer patients participating in trials [3, 5–10]. Our results show that several QLQ-C30 HRQoL scales and items, including the Summary Score, and some QLQ OG25 items, are significantly associated with OS in the real-world setting as well.

For potentially curable patients, GHS was an independent prognostic factor. GHS is one of the most used HRQoL endpoints in clinical trials within oesophagogastric cancer. In addition, four items of the QLQ-OG25, i.e., dysphagia, eating restrictions, odynophagia and body image, were independent prognostic factors for OS, highlighting the importance of the use of this questionnaire in addition to the QLQ-C30.

It could be argued that dysphagia, eating restrictions and odynophagia could be associated with tumour size and/or topography. However, these scores remained independent prognostic factors even when adjusting for clinical stage. Its specific relation to tumour size could not be investigated in our study population due to a lack of data on the precise size of the tumour, and possible mechanical obstruction. Interestingly, body image was also prognostic for OS, which is in line with recent results in pancreatic cancer patients [24]. It is hypothesized that body image is associated with nutritional status, and that involuntary weight loss resulting in cachexia may induce a negative perception of one’s body [25]. A strong association between cancer-associated weight loss and cachexia with OS has been observed in many cancer types [26–28], including in this POCOP population [29].

In patients with advanced oesophagogastric cancer, the Summary Score was independently associated with OS. The population-based study of Husson et al. also found that the Summary Score was a strong prognostic factor across several cancer types, with an overall HR of 0.77—which is comparable with the HR of 0.75 we observed [17]. Moreover, we found that pain was independently associated with OS. As argued by Mierzynska et al., patient reported pain might be more sensitive during specific disease stages than medical imaging results, indicating that pain could be indicative of progression even before growth could be measured by medical imaging techniques [3].

Whereas previous studies suggested that the prognostic value of HRQoL may vary across cancer types [2, 17], this study was focused on differences within one cancer type, i.e., between patients with potentially curable and advanced disease. We chose to divide our sample into these two patient groups because disease characteristics, treatment and survival differ substantially. Moreover, these differences may also impact HRQoL. Lastly, in clinical trials, there is often a clear distinction between patients with potentially curable and advanced disease.

In our study, we found the Summary Score to be an independent prognostic factor in the advanced disease subgroup, whereas GHS was found prognostic in potentially curable patients. In addition, dysphagia, eating restrictions, appetite loss, odynophagia, and body image had prognostic value in potentially curable patients, while role functioning, fatigue, insomnia and pain had prognostic value within advanced disease. While the prognostic HRQoL items in patients with advanced disease were more focused on symptoms beyond local tumour burden, it is expected that towards death symptom burden relating to other body functions increase, e.g., expressed as fatigue, insomnia, pain, and role functioning. In contrast, most prognostic HRQoL items in potentially curable patients are symptomatic for oesophagogastric functions – which is affirmed by its inclusion in the QLQ-OG25 cancer specific module. Given the borderline significant results of a subset of HRQoL variables, we also could argue that given the relatively small sample size (especially for patients with advanced disease) and a 1% alpha value, we reduced the risk of obtaining type 1 errors but increased the risk of type 2 errors. This could result in the failure to identify significant prognostic HRQoL variables. We recommend future studies to account for differing effect sizes per HRQoL item and longer follow-up for potentially curable patients, given our study was designed to detect HRs of 0.9 (GHS) to 0.85 (Summary Score) for potentially curable patients and 0.83 (GHS) to 0.77 (Summary Score) for patients with advanced disease.

Although physical functioning is one of the most reported prognostic domains of the QLQ-C30 across different cancer types [3], we did not find it to be an independent prognostic factor when adjusting for other variables, including treatment type and/or performance status. This might be due to multicollinearity between physical functioning, performance status, and received treatment. The same interrelationship may hold for dysphagia, eating restrictions, odynophagia, and appetite loss, which may explain why only eating restrictions was retained in the exploratory multivariable model. Even when several HRQoL items are separately prognostic for survival alongside clinical variables, multicollinearity issues may still arise given the potential co-occurrence and overlap of the symptoms included in the EORTC QLQ-OG25.

Strengths of this population-based study are its multicentre design, representing more than half of the hospitals in the Netherlands. The amount of missing data at the item level was very limited, mostly due to POCOP's design [18]. In our analyses, we tested the prognostic value of HRQoL alongside established prognostic clinical and sociodemographic factors, as recommended by Mierzynska et al. [3] Since clinical practice and decision making are mainly based on clinical, sociodemographic, and/or pathological information, we applied this approach to our analysis as well. Within this clinically driven perspective, we believe it is key to investigate the extent to which HRQoL can add additional information regarding prognostication. Regarding the additional explained variance in OS, only the Summary Score was found to explain > 5% of the survival outcome in patients with advanced disease. While a 5% threshold is somewhat arbitrary, our findings show that although statistically significant, the added value of most HRQoL scales are only modest. This finding is supported by other studies across a range of cancer types [3, 30]. It should be noted that when the additional explained variance in survival of clinical variables is assessed by addition to a HRQoL model, the explained variance of HRQoL variables would be somewhat higher.

Limitations of this study were included patients who filled out PROMS within seven days after starting initial treatment. Officially, these data are, therefore, not true baseline values. However, there was no statistically significant association between OS and HRQoL between patients with true and non-true baseline data. Absolute HRQoL and OS values were also comparable between these two groups. Therefore, we see no additional risk of bias. Additionally, we only included patients who were participating in POCOP and hence were willing to complete questionnaires. Our sample consisted of fewer patients (14%) with advanced disease in comparison to the population prevalence of advanced disease at diagnosis, which is 40–50% [31, 32]. This potential selection bias may, therefore, hamper the external validation of this study.

Given our results, it would be important to investigate what type of care could be offered to patients with lower HRQoL scores and to what extent this care would prolong survival. Within the POCOP database, specific interventions focused on improving HRQoL are not registered systematically and, therefore, it remains unknown how and to what extent patients within this cohort were supported. Interventions should preferably be patient-based and tailored to the individual needs, for example support provided by dieticians, psychologists, or physiotherapists. Recent research from Basch et al. showed that in patients with advanced cancer, real-time monitoring of patient reported symptoms can result in meaningful benefits, including a lesser decline in HRQoL over time, remaining longer on chemotherapy, and longer (quality-adjusted) survival [33]. Overall, these findings suggest that measuring HRQoL at baseline and during treatment as part of a standardized clinical care path, could provide benefit to patients. Further research could investigate to what extent the EORTC HRQoL questionnaires could be utilized, in which frequency, and what cut-off thresholds could be useful to guide supportive care activities.

## Conclusion

HRQoL was significantly associated with OS in patients with potentially curable and advanced oesophagogastric cancer in a real-world setting. The HRQoL items that were found to be prognostic, including the recently developed Summary Score and several OG25 items, could be used to develop or update prognostic models in oesophagogastric cancer.

## Supplementary Information

Below is the link to the electronic supplementary material.

Supplementary file1 (DOCX 19 KB)

## References

[1] Montazeri A (2009). Quality of life data as prognostic indicators of survival in cancer patients: An overview of the literature from 1982 to 2008. Health Qual Life Outcomes.

[2] Quinten C, Coens C, Mauer M, Comte S, Sprangers MAG, Cleeland C (2009). Baseline quality of life as a prognostic indicator of survival: a meta-analysis of individual patient data from EORTC clinical trials. Lancet Oncol.

[3] Mierzynska J, Piccinin C, Pe M, Martinelli F, Gotay C, Coens C (2019). Prognostic value of patient-reported outcomes from international randomised clinical trials on cancer: a systematic review. Lancet Oncol.

[4] Gotay CC, Kawamoto CT, Bottomley A, Efficace F (2008). The prognostic significance of patient-reported outcomes in cancer clinical trials. J Clin Oncol.

[5] ter Veer E, van Kleef JJ, Schokker S, van der Woude S, Laarman M, Haj Mohammad N (2018). Prognostic and predictive factors for overall survival in metastatic oesophagogastric cancer: A systematic review and meta-analysis. Eur J Cancer.

[6] Park SH, Cho MS, Kim YS, Hong J, Nam E, Park J (2008). Self-reported health-related quality of life predicts survival for patients with advanced gastric cancer treated with first-line chemotherapy. Qual Life Res.

[7] Blazeby JM, Brookes ST, Alderson D (2001). The prognostic value of quality of life scores during treatment for oesophageal cancer. Gut.

[8] Kidane B, Sulman J, Xu W, Kong QQ, Wong R, Knox JJ (2016). Baseline measure of health-related quality of life (Functional Assessment of Cancer Therapy-Esophagus) is associated with overall survival in patients with esophageal cancer Read at the 95th Annual Meeting of the American Association for Thoracic Surgery. J Thorac Cardiovasc Surg.

[9] Van Heijl M, Sprangers MAG, De Boer AGEM, Lagarde SM, Reitsma HB, Busch ORC (2010). Preoperative and early postoperative quality of life predict survival in potentially curable patients with esophageal cancer. Ann Surg Oncol.

[10] Bergquist H, Johnsson Å, Hammerlid E, Wenger U, Lundell L, Ruth M (2008). Factors predicting survival in patients with advanced oesophageal cancer: A prospective multicentre evaluation. Aliment Pharmacol Ther.

[11] Sherman RE, Anderson SA, Dal Pan GJ, Gray GW, Gross T, Hunter NL (2016). Real-World Evidence — What Is It and What Can It Tell Us?. N Engl J Med.

[12] Templeton AJ, Booth CM, Tannock IF (2020). Informing patients about expected outcomes: The efficacy-effectiveness gap. J Clin Oncol.

[13] Chau I, Norman AR, Cunningham D, Waters JS, Oates J, Ross PJ (2004). Multivariate prognostic factor analysis in locally advanced and metastatic esophago-gastric cancer - Pooled analysis from three multicenter, randomized, controlled trials using individual patient data. J Clin Oncol.

[14] Ter Veer E, van Kleef JJ, Sprangers MAG, Haj Mohammad N, van Oijen MGH, van Laarhoven HWM (2018). Reporting of health-related quality of life in randomized controlled trials involving palliative systemic therapy for esophagogastric cancer: a systematic review. Gastric Cancer.

[15] Lagergren P, Fayers P, Conroy T, Stein HJ, Sezer O, Hardwick R (2007). Clinical and psychometric validation of a questionnaire module, the EORTC QLQ-OG25, to assess health-related quality of life in patients with cancer of the oesophagus, the oesophago-gastric junction and the stomach. Eur J Cancer.

[16] Giesinger JM, Kieffer JM, Fayers PM, Groenvold M, Petersen MAA, Scott NW (2016). Replication and validation of higher order models demonstrated that a summary score for the EORTC QLQ-C30 is robust. J Clin Epidemiol.

[17] Husson O, Rooij BH, Kieffer J, Oerlemans S, Mols F, Aaronson NK (2020). The EORTC QLQ-C30 summary score as prognostic factor for survival of patients with cancer in the “Real-World”: Results from the population-based PROFILES registry. Oncologist.

[18] Coebergh van den Braak RRJ, van Rijssen LB, van Kleef JJ, Vink GR, Berbee M, van Berge Henegouwen MI, et al. Nationwide comprehensive gastro-intestinal cancer cohorts: the 3P initiative. Acta Oncol. 2018;57(2):195–202. 10.1080/0284186X.2017.1346381 28723307

[19] Van De Poll-Franse LV, Horevoorts N, van Eenbergen M, Denollet J, Roukema JA, Aaronson NK (2011). The patient reported outcomes following initial treatment and long term evaluation of survivorship registry: Scope, rationale and design of an infrastructure for the study of physical and psychosocial outcomes in cancer survivorship cohorts. Eur J Cancer.

[20] Fritz A, Percy C, Jack A, Shanmugaratnam K, Sobin L, Parkin DM, et al., editors. International Classification of Diseases for Oncology (ICD-O). 3rd Ed. Geneva: World Health Organisation; 2000.

[21] Fayers P, Bottomley A (2002). Quality of life research within the EORTC—the EORTC QLQ-C30. Eur J Cancer.

[22] Fayers PM, Aaronson NK, Bjordal K, Groenvold M, Curran D, Bottomley A, on behalf of the EORTC Quality of Life Group. The EORTC QLQ-C30 Scoring Manual (3rd Edition). Brussels, 2001.

[23] Osoba D (2011). Health-related quality of life and cancer clinical trials. Ther Adv Med Oncol.

[24] MacKay TM, Latenstein EJ, Sprangers MAG, van der Geest LG, Creemers GJ, van Dieren S (2020). Relationship between quality of life and survival in patients with pancreatic and periampullary cancer: A multicenter cohort analysis. JNCCN J Natl Compr Cancer Netw.

[25] Hopkinson JB (2014). Psychosocial impact of cancer cachexia. J Cachexia Sarcopenia Muscle.

[26] Martin L, Senesse P, Gioulbasanis I, Antoun S, Bozzetti F, Deans C (2015). Diagnostic criteria for the classification of cancer-associated weight loss. J Clin Oncol.

[27] Martin L, Birdsell L, MacDonald N, Reiman T, Clandinin MT, McCargar LJ (2013). Cancer cachexia in the age of obesity: Skeletal muscle depletion is a powerful prognostic factor, independent of body mass index. J Clin Oncol.

[28] Baracos VE, Martin L, Korc M, Guttridge DC, Fearon KCH (2018). Cancer-associated cachexia. Nat Rev Dis Prim.

[29] Dijksterhuis WPM, Latenstein AEJ, Van Kleef JJ, Verhoeven RHA, de Vries JHM, Slingerland M (2021). Cachexia and dietetic interventions in patients with esophagogastric cancer: A multicenter cohort study. JNCCN J Natl Compr Cancer Netw.

[30] Mauer MEL, Taphoorn MJB, Bottomley A, Coens C, Efficace F, Sanson M (2007). Prognostic value of health-related quality-of-life data in predicting survival in patients with anaplastic oligodendrogliomas, from a phase III EORTC brain cancer group study. J Clin Oncol.

[31] Nelen SD, van Putten M, Lemmens VEPP, Bosscha K, de Wilt JHW, Verhoeven RHA (2017). Effect of age on rates of palliative surgery and chemotherapy use in patients with locally advanced or metastatic gastric cancer. Br J Surg.

[32] van Putten M, de Vos-Geelen J, Nieuwenhuijzen GAP, Siersema PD, Lemmens VEPP, Rosman C (2018). Long-term survival improvement in oesophageal cancer in the Netherlands. Eur J Cancer.

[33] Basch E, Deal AM, Dueck AC, Scher HI, Kris MG, Hudis C (2017). Overall survival results of a trial assessing patient-reported outcomes for symptom monitoring during routine cancer treatment. JAMA.

