# Supplementary material for: Prognostic value of patient-reported quality of life for survival in oesophagogastric cancer: analysis from the population-based POCOP study
Source: Gastric Cancer. 2021 Jul 12;24(6):1203–12. doi: 10.1007/s10120-021-01209-1 (PMC8502147; doi:10.1007/s10120-021-01209-1)
Supplement: Supplementary file 1 — Supplementary file1 (DOCX 19 KB) [file 10120_2021_1209_MOESM1_ESM.docx]

**Online Resource 1:** Univariate cox regression analysis of the secondary HRQoL items of interest of the EORTC QLQ-C30 and QLQ-OG25.

| **Secondary HRQoL variables** | **Patients with potentially curable disease** | | **Patients with advanced disease** | |
| --- | --- | --- | --- | --- |
| **Univariate cox regression analysis** | **HR (99% CI)** | **p** | **HR (99% CI)** | **p** |
| Physical functioning | **0.90 (0.82-0.98)** | **0.001** | **0.82 (0.72-0.95)** | **<0.001** |
| Role functioning | **0.94 (0.89-1.00)** | **0.01** | **0.91 (0.83-1.00)** | **0.01** |
| Emotional functioning | 1.02 (0.94-1.10) | 0.59 | 0.94 (0.83-1.07) | 0.25 |
| Cognitive functioning | 1.00 (0.91-1.09) | 1.00 | 0.89 (0.76-1.04) | 0.05 |
| Social functioning | 0.94 (0.88-1.01) | 0.02 | 0.93 (0.84-1.03) | 0.08 |
| Fatigue | 1.06 (0.99-1.14) | 0.02 | **1.15 (1.02-1.29)** | **0.002** |
| Nausea and vomiting | **1.08 (1.00-1.16)** | **0.009** | **1.16 (1.03-1.31)** | **0.001** |
| Pain | **1.10 (1.02-1.19)** | **0.001** | 1.09 (0.97-1.22) | 0.06 |
| Dyspnea | 1.01 (0.94-1.09) | 0.69 | 1.05 (0.93-1.19) | 0.31 |
| Insomnia | 1.01 (0.95-1.07) | 0.71 | **1.10 (1.00-1.21)** | **0.007** |
| Appetite loss | **1.09 (1.04-1.15)** | **0.0001** | **1.10 (1.03-1.19)** | **0.001** |
| Constipation | 1.04 (0.97-1.12) | 0.13 | 1.02 (0.83-1.14) | 0.53 |
| Diarrhea | 1.02 (0.94-1.12) | 0.51 | 1.09 (0.95-1.26) | 0.09 |
| Financial difficulties | 0.98 (0.89-1.08) | 0.62 | 1.01 (0.86-1.18) | 0.91 |
| Dysphagia | **1.13 (1.07-1.21)** | **<0.001** | 1.06 (0.96-1.18) | 0.11 |
| Eating restrictions | **1.12 (1.06-1.18)** | **<0.001** | **1.09 (1.01-1.18)** | **0.005** |
| Reflux | 1.07 (0.99-1.15) | 0.02 | 1.11 (0.91-1.34) | 0.18 |
| Odynophagia | **1.08 (1.02-1.14)** | **<0.001** | 1.07 (0.97-1.19) | 0.07 |
| Pain and discomfort | 1.06 (0.99-1.12) | 0.02 | 1.09 (0.98-1.21) | 0.04 |
| Anxiety | 0.98 (0.93-1.04) | 0.46 | 1.02 (0.93-1.12) | 0.56 |
| Eating with others | 1.05 (1.00-1.10) | 0.02 | 1.04 (0.95-1.13) | 0.29 |
| Dry mouth | 1.05 (0.99-1.12) | 0.04 | **1.13 (1.04-1.24)** | **0.000** |
| Trouble with taste | **1.07 (1.01-1.13)** | **0.005** | 1.06 (0.97-1.16) | 0.07 |
| Body image | **1.09 (1.03-1.17)** | **<0.001** | **1.12 (1.02-1.23)** | **0.003** |
| Trouble swallowing saliva | 1.06 (0.99-1.13) | 0.02 | 1.08 (0.98-1.19) | 0.05 |
| Choked when swallowing | 1.04 (0.96-1.13) | 0.21 | 1.15 (0.93-1.3 | 0.04 |
| Coughing | 1.06 (0.99-1.13) | 0.02 | 1.05 (0.94-1.17) | 0.26 |
| Trouble talking | 1.07 (0.95-1.20) | 0.14 | 1.04 (0.90-1.22) | 0.52 |
| Worrying about weight loss | **1.07 (1.01-1.13)** | **0.002** | **1.10 (1.02-1.18)** | **0.002** |
| *HRQoL* health-related quality of life, *HR* hazard ratio. Hazard ratios are given for every 10-point increase in HRQoL scores. Values in bold were statistically significant at p<0.01. | | | | |
